# Challenges in the Management of Gram-Negative Bacterial Infections in Patients With Ventriculoperitoneal Shunt

**DOI:** 10.7759/cureus.17035

**Published:** 2021-08-09

**Authors:** Faran Ahmad, Margaret Brubaker, Sanu S Rajendraprasad, Brian Hoeynck, Brent L Clyde, Manasa Velagapudi

**Affiliations:** 1 Infectious Diseases - Critical Care Medicine, Creighton University School of Medicine, Omaha, USA; 2 Internal Medicine, University of Cincinnati Medical Center, Cincinnati, USA; 3 Internal Medicine, Creighton University School of Medicine, Omaha, USA; 4 Internal Medicine, Creighton University, Omaha, USA; 5 Internal Medicine, University of Pennsylvania, Philadelphia, USA; 6 Neurosurgery, Creighton University School of Medicine, Omaha, USA; 7 Infectious Diseases, Creighton University School of Medicine, Omaha, USA

**Keywords:** gram-negative meningitis, cerebrospinal fluid (csf), cerebrospinal fluid shunt, ventriculoperitoneal shunt placement, keywords: nosocomial infections

## Abstract

Gram-negative bacterial infections of the central nervous system (CNS) have worse clinical outcomes. The most common bacteria include *Escherichia Coli*, Citrobacter species, Enterobacter species, Serratia species, and *Pseudomonas aeruginosa*. There are multiple risk factors for CNS infection after shunt insertion, including younger age, obstructive hydrocephalus, shunt revision surgery, and trauma. The clinical presentation of a ventriculoperitoneal (VP) shunt infection includes the signs and symptoms of meningitis to fever with abdominal pain and peritonitis. Apart from cerebrospinal fluid (CSF) analysis, microbiological cultures and radiological studies are key diagnostic tools. Initial empirical intravenous antimicrobial therapy is preferably broad spectrum with appropriate coverage for resistant Gram-negative pathogens and the duration of treatment depends upon pathogenesis, host factors, and clinical response to the therapy.

Considering the importance of this disease and associated clinical outcomes, in this review article, we have summarized the epidemiology, clinical features, management, and prevention of Gram-negative VP shunt infections in adults.

## Introduction and background

Introduction

Cerebrospinal fluid (CSF) infections and ventriculitis caused by Gram-negative bacteria have worse outcomes [1]. The purpose of this review article is to discuss the epidemiology, clinical features, management, and prevention of Gram-negative ventriculoperitoneal (VP) shunts in adults.

We conducted a systematic search of the PubMed, MEDLINE, and Google Scholar databases from 1972 to 2020. All articles in English were reviewed. For PubMed, we searched for the articles and titles with Gram-negative bacteria, Gram-negative bacterial infections, CSF, and VP shunts. For MEDLINE and Google Scholar, we searched titles and abstracts containing the words CSF and VP shunt and Gram-negative bacterial infections.

Background

The incidence of CSF shunt infections widely varies from 1% to 30%, and up to 35% of the infected shunts had Gram-negative bacteria as a pathogen [1].

Over the six-year follow-up, Lee et al. (2012) showed that 35 out of 333 (10.5%) shunts became infected. Four of those infections were secondary to Gram-negative bacteria (1.2%) [2]. Meanwhile, a retrospective study by Ochieng et al. published in 2015 showed that the incidence of Gram-negative bacteria causing VP shunt infections is probably higher outside the United States. In this study, 39.6% of Kenyan children with VP shunt infections were infected with Gram-negative bacteria; most of which were multidrug-resistant [3]. However, additional studies are required to determine if other Sub-Saharan countries have a higher incidence of Gram-negative bacterial VP shunt infections as compared to the United States.

The possibility of ventriculoperitoneal (VP) shunt infection is most likely to be in the earlier days after shunt insertion. Between 56% and 87% of infections occur within one month of shunt insertion [2-3]. Further risk factors for VP shunt infection include the expertise of the neurosurgeon, postoperative CSF leakage, and the use of a neuro-endoscope [4]. A 20-year longitudinal study showed that the infection rate at six months was 5.6% and increased (cumulatively) to 6.4%, 8.0%, 9.0%, and 10.0% in one, 5, 10, and 20 years after shunt placement surgical procedure, respectively [5].

Gender and age are additional risk factors for VP shunt infections. The infection rate was much higher in pediatric patients (age < 17 years) compared with adult patients (21.3% vs. 5.9%). Furthermore, VP shunt insertion before one year of age is independently associated with a higher risk of VP shunt infections. There is some data to suggest that the odds of infection among male patients are 1.67 times higher than for female patients, however, the reasons for the difference are not clear [5].

The underlying conditions commonly associated with VP shunt infections include congenital hydrocephalus (24.8%) and spinal dysraphism (23.1%). The infection rate is higher with obstructive hydrocephalus (13.1%) compared to communicating hydrocephalus (6.5%) or idiopathic hydrocephalus (2.5%). Other etiologies with lower risk for infections include cerebral hemorrhage (11.9%), trauma (8.6%), post craniotomy (6.7%), and tumors/cysts (6.1%). The odds of infection are 1.94 times higher among patients with high-risk etiologies (such as congenital hydrocephalus and spinal dysraphism) than those with medium or low-risk etiologies [5]. Shunt revision surgery also poses considerable risk of infection. A study in 2012 found a higher infection rate (17.2%) after shunt re-insertion following explantation due to infection as compared to initial shunt placement (8.9%). This difference is considerable because approximately half of all the patients with VP shunt placements require at least one surgical revision [6].

In cases of bowel perforation or patients with myelomeningocele who have undergone multiple intra-abdominal procedures related to either bowel or bladder incontinence, retrograde infection from the distal end of a VP shunt is considered as a reasonable risk factor for shunt infection [7]. Further research is needed to determine additional risk factors that predispose to Gram-negative VP shunt infections.

## Review

There is a wide spectrum of clinical manifestations secondary to VP shunt infections. This may include non-specific symptoms of fever and abdominal pain in addition to local signs and symptoms of shunt infections, including tenderness and erythema of the scalp overlying the shunt tubing [8]. Also, shunt malfunction may result in increased intracranial pressure or meningitis and hence cause associated clinical symptoms. In a retrospective review of 32 patients with shunt infections, 89% of the patients were febrile at the time of presentation [9]. However, local shunt tract pain and/or signs of shunt malfunction can present without fever in patients with shunt malfunction.

Interestingly, abdominal tenderness, guarding and rigidity may develop in patients with VP shunt who develop peritonitis [10]. Abdominal pseudocysts at the distal end of the shunt can cause partial or complete shunt obstruction [11]. These patients usually present with an unexplained occlusion of the peritoneal catheter or failure of peritoneal CSF absorption.

Manifestations of a VP shunt infection can relate to increased intracranial pressure, ventriculitis, or meningitis. This may manifest as neck stiffness, altered mentation or neurologic function, headache, and nausea. This presentation is often related to shunt obstruction caused by infection. This type of presentation was seen in less than 50% of cases. Local symptoms, such as erythema, pain, and purulence, may be present along the course of the shunt, although this is an inconsistent finding, and were present in 49% of cases in the retrospective analysis and 26% in the review article [9]. Clinically, individual symptoms are not typically both sensitive and specific [10-11]. Because most shunt infections occur in the first few months after shunt surgery, the positive and negative predictive values of individual symptoms will depend on when the shunt procedure was performed [11-12].

Diagnostic criteria

The United States Center for Disease Control and Prevention’s National Healthcare Safety Network has decided that meningitis or ventriculitis must meet at least one of the following criteria [13-14]:

1. Isolation and identification of the pathogenic organism(s) from CSF by microbiologic testing methods, including culture or non-culture-based diagnostic modalities, which are not performed for surveillance purposes.

2. The patient should have at least two of the following: i. Fever (>38.0°C) or headache; and ii. Meningeal sign(s); iii. Cranial nerve sign(s).

And at least one of the following: a. Low CSF glucose levels, elevated CSF white cells, and increased CSF protein levels; b. Gram stain of CSF showing the suspected organism(s); c. Isolation and identification of the pathogenic organism(s) from the blood by microbiologic testing methods, including culture or non-culture-based diagnostic modalities, which are not performed for surveillance purposes; d. A 4-fold increase in paired sera (IgG) for the organism or a diagnostic single IgM antibody titer.

Cerebrospinal Fluid Analysis

Shunt fluid evaluation is simple, relatively low risk, and is the definitive study for diagnosing a shunt malfunction and/or shunt-related infection [15]. To confirm the diagnosis, either a direct culture of the CSF obtained by shunt aspiration or by culture of explanted shunt hardware is required. VP shunt fluid is usually obtained from a reservoir, which is typically in an easily accessible location, subcutaneously near or incorporated into the shunt valve. After sterile preparation, the reservoir should be punctured with a 25-gauge needle. The risk of introducing an infection should be considered with a shunt tap but is reported to be low (4.1%) [16]. On occasion, the shunt may be tapped for evaluation of shunt function in patients with no clinical signs of infection and may be found to be culture-positive. In this situation, one must determine if this is contamination or infection. Depending on the clinical situation, the shunt might need to be re-tapped and a positive culture with the same microorganisms would indicate true infection. Meanwhile, a contaminating microorganism is defined as an isolated positive CSF culture and/or positive Gram stain with a normal CSF count, CSF glucose, and CSF protein [15]. A 10-day observation period for CSF specimens can be justified [17].

Laboratory Tests

CSF should be sent for cell count with differential, glucose and protein, Gram stain, and aerobic and anaerobic cultures. Gram-negative organisms had higher initial and peak WBC count with a greater differential of polymorphonuclear (PMN) leukocytes compared with other organisms. There is an initial predominance of PMN leukocytes, followed by a delayed peak of lymphocytes, monocytes, and eosinophils over a 14-day course. All values trended toward zero over the treatment course [18]. CSF lactate has high sensitivity and specificity in differentiating bacterial from viral meningitis. CSF lactate has increased diagnostic accuracy for bacterial meningitis using 3 mmol/L as the cut-off, whereas in the case of viral meningitis, the CSF lactate remains below 2 mmol/L [19-20]. CSF lactate and serum procalcitonin elevations are also associated with other processes and thus should be considered in the context of the overall clinical presentation and lab data [21].

While the gold standard for diagnosis has been CSF culture, the sensitivity has been ≤80% [22]. Bacterial pathogens were detected by 16S rRNA PCR in CSF samples of patients with bacterial meningitis with excellent sensitivity and specificity. The film array meningitis/encephalitis (ME) panel had a higher sensitivity than CSF culture in detecting almost all bacteria, although *Escherichia coli* *K1*, *Haemophilus influenzae*, and *Neisseria meningitidis* are the only Gram-negative organisms that can be detected in the current panel [23]. Furthermore, real-time PCR and Gram staining are less affected by the antibiotic presence and might be useful when antibiotics were previously administered.

Cultures

CSF culture from the shunt, reservoir, or drain is the most important test to establish the diagnosis of CSF infection. In patients with an infected VP shunt, the yield of a positive culture is greater when CSF is obtained from a shunt aspiration (91-92%) than from CSF obtained from lumbar puncture (45-67%) [15]. The culture will usually be positive if the device is infected even when there is no pleocytosis or alteration in CSF chemistries. CSF cultures may require several (3-10) days to weeks of incubation before they can be called negative, especially in patients who received previous antimicrobial therapy.

Imaging

Plain radiography can be helpful if shunt malfunction is suspected to rule out disconnection or misplaced distal hardware. Retained hardware that could be a source for ongoing infection can also be seen on plain radiography, though removing such tubing is generally difficult to do completely. Shunt obstructions can be confirmed with radioisotope studies or with the injection of iodinated contrast material into the shunt reservoir with fluoroscopically visualized runoff [24]. Ventriculitis and meningitis can sometimes be visualized with contrast-enhanced computed tomography (CT) and magnetic resonance imaging (MRI) as enhancement of the ventricular ependymal lining or cerebral cortical sulci. MRI with gadolinium and diffusion-weighted imaging is the most useful modality since it is more sensitive than CT for detecting ventriculitis. Fluid attenuation inversion recovery and post-contrast T-1 weighted images are particularly useful. Diffusion-weighted imaging may be used to detect pus in the ventricular system (visualized as a bright signal) and to differentiate a brain abscess from malignancy [25]. F-18 fluorodeoxyglucose (FDG) position emission tomography (PET) computed tomography (CT) is an accepted infection imaging tool and can be used to diagnose shunt infection accurately in a patient with high clinical suspicion [25-26].

Pseudocysts are loculated collections of CSF that form around the distal end of the catheter in the peritoneum. Pseudocyst formation is a common cause of distal catheter obstruction [24]. Pseudocysts are caused by peritoneal adhesion or migration of the greater omentum over the shunt tip. A definitive diagnosis can be made by abdominal CT or ultrasound showing a cystic fluid collection surrounding the distal catheter tip [25].

Treatment

The treatment principles of Gram-negative VP shunt-associated ventriculitis are like those considered in selecting antimicrobial therapy for acute bacterial meningitis. These include CNS penetration of the antibiotics, the therapeutic concentration at the site of infection, adequate bactericidal activity, and minimizing systemic side effects of the treatment dose of antibiotics [27]. In appropriate clinical settings when a CSF pleocytosis is present, empirical antibiotic therapy should be started after appropriate cultures have been collected.

The selection of an appropriate empirical antibiotic regimen depends upon the pathogenesis of the infection and associated factors, including neurosurgical procedure, prolonged hospital course after head trauma, previous microbiological data, local antimicrobial susceptibility pattern, and recent exposure to extended-spectrum antibiotics [28]. The most likely Gram-negative bacilli associated with VP shunt-associated ventriculitis include *Escherichia coli*, Citrobacter species, Enterobacter species, Serratia species, and *Pseudomonas aeruginosa*. Initial empirical therapy should be broad-spectrum, with appropriate coverage for resistant Gram-negative pathogens, including cefepime, ceftazidime, or meropenem. Meropenem is used in patients with beta-lactam allergy. Also, intravenous meropenem is preferred due to the lower risk of seizure compared to imipenem, and clinical studies have shown its benefit in the empirical treatment of bacterial meningitis [29-31]. Once the Gram-negative organism is identified, antibiotics can be switched to pathogen-specific therapy. In patients who cannot tolerate or have a contraindication to carbapenems, aztreonam or ciprofloxacin can be used as an alternative (Table 1).

**Table 1:** Preferred antimicrobial therapies for targeted treatment ESBL: extended spectrum beta-lactamases

Organism	Recommended treatment	Alternative therapy
Susceptible Gram-negative bacilli	Ceftriaxone or cefotaxime	
Pseudomonas species	Cefepime, ceftazidime, or meropenem	Aztreonam or fluoroquinolone
ESBL- Gram-negative bacilli	Meropenem	Cefepime or a fluoroquinolone
Acinetobacter species	Meropenem	Colistimethate sodium or polymyxin B

Removal of the infected shunt results in rapid clearance of infections, as certain microorganisms have the potential to adhere and form a biofilm on the catheters, i.e. *Pseudomonas aeruginosa* [6]. Similarly, once infected, removal of all components of the infected internal ventricular catheters along with targeted antimicrobial therapy results in treatment success in up to 85% of the patients [28]. In case of clinical necessity (monitoring of CSF findings, culture, and treatment of hydrocephalus), placement of a temporary external ventricular drain can be considered, before replacing the long-term VP shunt [28].

Most studies on VP shunt ventriculitis are either pharmacokinetic studies or uncontrolled case series [32]. Adequate doses of each antimicrobial therapy are described separately (Table 2).

**Table 2:** Total daily dose (dosing interval in hours)

Antimicrobial agent	Infants and children	Adults
Cefepime	150 mg/kg (8)	6 g (8)
Cefotaxime	300 mg/kg (6–8)	8–12 g (4–6)
Meropenem	120 mg/kg (8)	6 g (8)
Rifampin	20 mg/kg (24)	600 mg (24)

The duration of therapy depends upon pathogenesis, host factors, and clinical response to the therapy. If there is significant CSF pleocytosis, low CSF glucose and associated systemic features, the duration of therapy should be at least 14 days. In cases with repeat isolation of the Gram-negative organism from CSF cultures, treatment should be extended to 10-14 days from the last positive culture. In these cases, if a new VP shunt re-implant is planned, it should be delayed for 10 days from the last negative CSF culture [8].

Intraventricular administration of antibiotics remains a topic of discussion. Considering insufficient evidence, the US Food and Drug Administration has not approved intraventricular antibiotic use. However, it has been recommended by the “Neurosurgery Working Party of the British Society for Antimicrobial Chemotherapy” to consider it in neurosurgical patients with postoperative meningitis and inadequate clinical improvement with intravenous antibiotics [33]. Due to the risk of neurotoxicity, particularly seizures, penicillin and cephalosporins should not be given intrathecally. Similarly, intraventricular antimicrobial agents are not recommended in infants based on data in a Cochrane review [34].

However, intraventricular administration of polymyxin B, colistimethate sodium, and gentamicin have been elaborated in various systematic reviews in adults [35]. The gentamicin dose is 4-8 mg in adults and 1-2 mg in children. Dose frequency depends upon drain output over 24 hours (if <50 mL/24 hours: every third day, 50-100 mL/24 hours: every second day, and if 100-150 mL/24 hours: once daily) [8].

The presence of a pseudocyst in the setting of any signs of shunt dysfunction is an indication to externalize the distal end of the shunt and follow CSF parameters from the externalized distal end. After the pseudocyst has resolved and any infection treated, the distal end can be re-internalized [36].

Prognosis and outcomes

Gram-negative VP shunt infections historically are reported to be associated with significant mortality and poor outcomes [37]. More recent studies by Stamos et al. included 23 children with Gram-negative VP shunt infections; none of them died and 87% of them recovered without any residual neurological sequelae. The decreased mortality and better outcomes in this study were attributed to prompt shunt removal with external ventricular drain placement and aggressive antimicrobial administration. Mortality varied with an etiologic agent too. *E. coli *VP shunt meningitis was reported to have less mortality than other Gram-negatives [38]. Peak CSF concentration of ceftriaxone has been noted to be 100-fold greater than the minimum inhibitory concentration of Gram-negative organisms, hence it has been shown to result in the rapid eradication of bacteria from CSF as compared to aminoglycosides, which failed to obtain a consistent CSF concentration; therefore, ceftriaxone has been shown to achieve rapid sterilization of CSF [39]. A meticulous and sterile surgical technique (including topical antiseptic), shorter procedure times, and perioperative antibiotic prophylaxis are key to prevent VP shunt infections in general [40].

## Conclusions

Gram-negative bacterial VP shunt infections are significant, as they are associated with worse clinical outcomes. Keeping in view the risk factors and trigger events, the treatment modalities may change. A combination of appropriate antimicrobial therapy, timely source control, and a multi-disciplinary approach, including infectious disease consult services, are required to effectively manage complex shunt infections and hence avoid the associated complications.
